# Sociohistorical Analysis of Normative Standards of Masculinity in the Pandemic of COVID-19: Impacts on Men’s Health/Mental Health

**DOI:** 10.3389/fpsyg.2022.775337

**Published:** 2022-05-11

**Authors:** Anderson Reis de Sousa, Wanderson Carneiro Moreira, Thiago da Silva Santana, Isabella Félix Meira Araújo, Cléa Conceição Leal Borges, Éric Santos Almeida, Magno Conceição das Mercês, Richardson Augusto Rosendo da Silva, Jules Ramon Brito Teixeira, Luciano Garcia Lourenção, Nadirlene Pereira Gomes, Evanilda Souza de Santana Carvalho, Álvaro Francisco Lopes de Sousa, Lílian Conceição Guimarães de Almeida, Larissa Vanessa Machado Viana, Álvaro Pereira

**Affiliations:** ^1^Escola de Enfermagem, Universidade Federal da Bahia (UFBA), Salvador, Brazil; ^2^Escola de Enfermagem, Universidade de São Paulo (USP), São Paulo, Brazil; ^3^Departamento de Saúde, Universidade Estadual de Feira de Santana (UEFS), Feira de Santana, Brazil; ^4^Departamento de Ciências da Vida, Universidade do Estado da Bahia (UNEB), Salvador, Brazil; ^5^Departamento de Enfermagem, Universidade Federal do Rio Grande do Norte (UFRN), Natal, Brazil; ^6^Escola de Enfermagem, Universidade Federal do Rio Grande (FURG), Rio Grande, Brazil; ^7^Escola de Enfermagem de Ribeirão Preto, Universidade de São Paulo (USP), São Paulo, Brazil; ^8^Global Heath and Tropical Medicine, Instituto de Higiene e Medicina Tropical, Universidade NOVA de Lisboa, Lisboa, Portugal; ^9^Centro de Ciências da Saúde e do Desporto, Universidade Federal do Acre (UFAC), Rio Branco, Brazil

**Keywords:** pandemics, COVID-19, masculinities, men’s health, mental health care, mental health

## Abstract

**Objective:**

This study aims to analyze sociohistorically how the normative patterns of hegemonic masculinity produced impacts on men’s health/mental health in the context of the COVID-19 pandemic.

**Methods:**

A qualitative study from a socio-historical perspective was conducted with 50 men based on an online survey. A semistructured form was applied. The data were analyzed by the Collective Subject Discourse method, interpreted in the light of the context of epidemic disease and hegemonic masculinity.

**Results:**

The experience of the pandemic exposed the normative patterns of masculinities from the consummation of acts representative of the pandemic context, which incited men to deny the existence of COVID-19 disease and to delay the understanding and adoption of measures to protect and control COVID-19. As a repercussion, men presented conflicts in the regulation of emotions; presented emotional suppression; were more reactive; felt threatened regarding the loss of the role of family provider, virility; and revealed a sense of invulnerability, added to the weakening of self-care.

**Conclusion:**

The discourse revealed that the men’s behaviors are consistent with the characteristics of hegemonic masculinity, but express signs of recognition that this behavior causes harm to themselves and their health.

## Introduction

The coronavirus disease 2019 (COVID-19) pandemic has been configured as a complex, multifaceted phenomenon of global magnitude (Guan et al., 2020). It currently represents the most complex health challenge of the century, causing repercussions for the living condition and health situation of populations, especially those in a greater context of vulnerability (Freitas et al., 2020; Nicola et al., 2020). It has been observed that men have presented the highest rates of contamination by the new coronavirus and have been more affected by COVID-19, even developing the most severe form of the disease, such as severe acute respiratory syndrome, as it occurs in Brazil (Coronavirus Epidemiological Bulletin 36, 2020).

In a sociohistorical way, it is possible to locate the phenomena characteristic of the pandemic that explain various repercussions, which can be analyzed from the identification of acts that compose a play and that can be framed as a picture (Rosenberg, C., 2010; Rosenberg and Mantovani, 2016). These sociohistorical phenomena are structured in a conjuncture way and influence the social fabric, like the social constructions of masculinities (Rosenberg, C. E., 2010; Connell and Messerschmidt, 2013). This theory constitutes a relevant milestone for the deepening of qualitative studies, in overcoming reductionisms about empiric and idealistic conceptions, in understanding the individuality of the subject, its relationship with the external—social and with historical processes, in this particular case, the pandemic of COVID-19.

The definition of normative patterns of masculinities constitutes the contours of hegemonic masculinity models, which use rigid attributes of definition of the human being in society, namely, heterosexuality, whiteness, wealth, dominance, power, subordination, and others that are directed to the maintenance of man in centrality and not in the periphery (Connell, 2005, 2014; Connell and Messerschmidt, 2005, 2013). However, tensions in this way have been provoked over the years by denouncing the potential for toxicity of this model for men themselves, for women and children, and for society—social organization (Connell, 2014).

Masculine and masculinity are directed to an empirical meaning, permeated by labels, objects, events, and specific qualities, in which, depending on culture, they are perceived and associated with men. It is important to add that the adjectives and nouns used to define masculinities are also used as a relevant theoretical construction of analysis of the social scene and fundamental to the self-concept of individuals (Spence, 1984). It is therefore indispensable to understand complex social phenomena, such as a pandemic, and other contexts that surround it, such as paternities, homosexuality, gender stereotypes, violence and male domination, corporeity, well-being, gender and health relations, already identified since the 1980s (Nolasco, 1981; Taylo, 1983; Bourdieu, 1999; Courtenay, 2000; Luck et al., 2000; Keijzer, 2003).

Hegemonic masculinity is a unique, essentialist, rigid model based on stereotyped and solidified gender characteristics and typologies. This model implies standardization, exclusion, and oppression of other models of non-dominant masculinity, which do not privilege heterosexuality, whiteness, eurocentrism, patriarchy, power, and the dominance of social class (Connell and Messerschmidt, 2013).

In this sense, it is justified to carry out studies that are dedicated to analyzing hegemonic masculinities and other emerging models of masculinities and the context of health, in order to locate tensions and identify flexibilities and discursive reformulations, transformations, and masculine movements toward the mobilization of masculinities to the ideas that are multiple, multidirectional, non-hierarchical, and plural, as already identified in the pandemic context (Connell and Messerschmidt, 2013; Jewkes et al., 2015; Sousa et al., 2021).

With the advent of the COVID-19 pandemic, gender relational aspects have been raised in the literature (Schurz et al., 2019; Sousa A. F. L. et al., 2020), not only to present the markers of gender and gender differentiation (Jin et al., 2020; Purdie, 2020) implicated in the onset of epidemic disease but also to explain questions related to how normative patterns of hegemonic masculinity can impact men’s life and health (Sanchez et al., 2020; Santos et al., 2020).

Given the health emergency of COVID-19, added to the need to deepen scientific knowledge about a new sociohistorical phenomenon, and the relevance in investigating the experiences of men living in Brazil regarding the health situation, the reasons for the development of this study are justified. Given the context presented, this study was guided by the research question: How did the normative standards of masculinity produce health/mental health impacts for men in the context of the COVID-19 pandemic? This article aims to analyze sociohistorically how the normative patterns of hegemonic masculinity produced impacts on men’s health/mental health in the context of the COVID-19 pandemic.

## Methods

This study is a qualitative study from a sociohistorical perspective. In this approach, the understanding of phenomena occurs from their historical event, in which the particular is considered an instance of the social and collective totality (Freitas et al., 2015). The research was conducted remotely in all regions of the country (e.g., north, northeast, central, southeast, and south). The participants of the research were 50 men, who met the following inclusion criteria: being a man and having legal majority—age equal to or above 18 years, and men who were declared not to have a fixed residence in Brazil. Foreign men who were in transit on international trips, who did not reside in Brazil, and who did not experience the daily epidemic of COVID-19 in Brazil were excluded from the study.

For the selection and recruitment of participants, we adopted the snowball technique (Patias and Hohendorff, 2019) applied in spaces of virtual ambience in digital social networks such as Facebook, Instagram, and WhatsApp. The technique used was supervised by four researchers with expertise and training in the area. All performed professional teaching and research activities during the data collection period; two were male researchers and two were female researchers. They had a direct relationship with the object of the study but had no previous connection with the participants because it was an online survey. For sample seizure, the theoretical saturation criterion of the data was considered (Nascimento et al., 2018).

The data collection occurred between April and June 2020 in a non-sequential and non-consecutive way between the states using Google Forms; they were validated internally by researchers and members of the research group and externally through a pilot test with 20 participants. A shape and terminologies were changed, without the need to adjust the content. The form consisted of two parts: Initially, closed questions, referring to sociodemographic characteristics, such as education, age, gender identity, sexual orientation, race/color, region of the country, type of housing, and with whom you live; employment, such as occupation and income; and health as a disease by COVID-19, access to the health system and professionals, and use of psychotropic drugs. Finally, to meet the objective of the study, the researchers proposed the following open questions: how did you experience the COVID-19 pandemic? As a man, have you noticed any impairments caused by the COVID-19 pandemic? These questions were selected from the previous analysis of the content on the theme published in the common media at the beginning of the pandemic and were derived from the discussions of the research group.

Participants’ responses were organized and systematized after reading line by line, processed and coded in the NVIVO12 software, and submitted to analysis by the Collective Subject Discourse (CSD) method (Lefevre et al., 2009). The procedure took place under the execution and supervision of researchers with expertise and training in the area. The total data obtained from the 50 forms were analyzed by identifying co-occurrences, convergences, and complementarity in compliance with the criteria of theoretical saturation (Nascimento et al., 2018) and COREQ guidelines (Pinto et al., 2018). These synthesis discourses start from the individual fragments of each participant and express social thought, that is, the opinion of the collectivity of the group of men investigated with the inclusion of their impressions, observations, and analysis about the phenomenon, materialized in textual writing (Lefevre and Lefevre, 2014).

The interpretation was based on the theories of Charles (Rosenberg, 1989, 1992, 2003; Rosenberg and Goldem, 1997; Rosenberg, C. E., 2010) who propose that the context of epidemic disease is organized into four representative acts, namely, progressive revelation, randomness management, negotiation of the public response, and subsidence and retrospection.

This perspective considers that the pandemic event is explained to the metaphor of framing, in an exercise of buying epidemics to plays and their paintings and four acts, which compose it. Therefore, it was possible to describe previous pandemic events with detail to locate existing dramaturgic acts at each time of the epidemiological course of diseases and analyze the impacts generated on the daily life of societies (Motta, 2020; Oliveira, 2020; Ramos Filho, 2020).

Moreover, for interpretative analysis of masculinities, the findings are anchored in the reference of masculinities, in the theoretical perspective proposed by Connell, which analytically defines the concept of masculinities and advances in scientific knowledge by revealing the concept of hegemonic masculinity and subaltern and/or marginalized masculinities (Connell, 2005, 2014; Connell and Messerschmidt, 2013). In addition, we used other references from the scientific literature on masculinities, as a way to enhance the scope of the discussion on the subject.

This study met all national and international standards of ethics in research involving human beings. The anonymity of the participants was ensured, with the identification of the discourses by the initials CSD (Collective Subject Discourse). The Research Ethics Committee approved the project, under the opinion of number CAAE: 32889420.9.0000.5531 and n. 4.087.611. This study was extracted from a Ph.D. thesis in Nursing and Health, linked to the School of Nursing and Health of the Federal University of Bahia, Brazil.

## Results

The characterization of the participants was delineated because they mostly had male gender identity—cisgender, gay sexual orientation, in the age group between 18 and 67 years, with self-declared brown race/color, complete higher education level, and more prevalent residence in the Northeast region in Brazil. They claimed to reside in masonry houses, with more than five rooms, in the coexistence of non-elderly relatives. The approximate income declared was more than five minimum wages.

Most of the participants worked and had formal work links. They made priority use of the private health system. Most did not seek support or support to cope with the pandemic; however, those who sought it chose to turn to family and friends and security and public administration services, and, finally, health services.

They are concerned about the pandemic scenario, whose most significant reasons for concern were the economic situation, the distancing of people from social life, and the situation of their loving relationships. They reported performing strategies to cope with the pandemic, such as the practice of leisure activities, hand washing and hygiene, use of alcohol gel, body hygiene, and compliance with quarantine. They pointed out that the pandemic brought changes in daily habits such as healthcare and increased habits such as consumption of content in the media and abusive consumption of alcohol and other drugs. Among the participants investigated, 18 reported having been diagnosed with COVID-19.

The findings that make up the “synthesis discourses” are anchored in the hegemonic masculinity constructs, which permeate the construction of the masculine in the discourse of men who reside in Brazil and experience the context of the COVID-19 pandemic in their country.

## Synthesis Discourses: Deleterious Impacts of Hegemonic Masculinity on Men’s Health in the Context of Pandemic

The categories emanating from the “Central Ideas” reflect the dimensions of the hegemonic patterns of masculinities tensioned with the advent of the pandemic and the deleterious impacts on health. The discourses are didactically framed in the theoretical context of the disease through the presentation of the contexts experienced by men in each figurative act proposed in the theory. The Central Ideas presented in the categories of Synthesium Discourses express most of the public investigated; however, the findings revealed the collective representation of this group.

### Central Idea 1: In COVID’s Progressive Revelation: Conflicts in the Regulation of Emotions and Suppression of Feelings

This category presents the first act of presentation of the Covid-19 pandemic, which takes place with a progressive revelation, in the face of emotional deflagration:

*[…] I have been feeling confused about my feelings most of the time since the pandemic arrived when it extended, I began to experience feelings of guilt, fear, anger and it affects me a lot and causes my mental health to be compromised. In the most of the time I keep these feelings and do not put out, hide and do not share with people and the fact that I spend more time indoors in isolation, in the absence of physical activities, without access to sunlight and contact with people and the fear of the consequences that may occur, has left me stressed and with mood swings. The fear was greater of being unemployed than of being infected and idleness has made me more reactive and introspective. I am a man and because of this, create barriers to expose emotions. Even before the pandemic I already had difficulty sharing feelings, anguish and fears and chose to keep myself quiet to comply with the “male” posture, rather than taking over and talking about my pains. Men tend to be more closed in relation to their feelings and being isolation at home increases the negative feelings, difficult to be dealt with. With the pandemic my communication became more limited I felt paralyzed. We are often taught how to be a provider, one who solves all situations all the time, but who does not learn to deal with his feelings. This situation is serious, because like me, other men may experience mental disorders such as depression, panic syndrome and anxiety disorders, which can lead to suicide* (Men’s CSD).

### Central Idea 2: In COVID-19 Randomness Management: Family Provision Compromise

The fear of consequences to family provision was evidenced in the collective discourse of men in the context of the pandemic:

*[…] I need to maintain the financial situation, take care of family members and other dependents, and this has generated frustration for not achieving these expectations. Many men like me are suffering in this pandemic because they are not being able to support the house, given that this is an assignment passed through several generations and involves a very great social pressure on the responsibilities to be fulfilled by me, and the male population. Now with the pandemic I feel that this pressure on having to provide for the family has become more aggravated, affecting my masculinity in various ways, whether it’s the pressure suffered in the workplace, which was exclusively a male environment, or for the family, which charged for me to manage and perform* (Men’s CSD).

### Central Idea 3: In COVID-19 Randomness Management: Threats to Virility

The fear of the emergence of threats to male virility was evidenced in the collective discourse of men in the context of the pandemic:

*[…] I am not being able to leave the house to deposit my energies in what I was doing before the pandemic, I have now felt more limited, needy and affected by the fact that I am single, thirsty for sex and not being able to perform it, due to isolation. I imagine this situation should also be happening to women, but this is a more common issue among men* (Men’s CSD).

### Central Idea 4: In Negotiation of the Public Response: Sense of Invulnerability and Adoption of Harmful Behaviors

The fragment discourse below revealed the exposure of the sense of male invulnerability in relation to the COVID-19. In addition, it expressed the adoption of harmful health behaviors adopted by men in the context of the pandemic:

*[…] sometimes I don’t feel so fragile and it’s also because men feel powerful to the point where they think it’s a cold, which ends up influencing me. I confess that we men tend to live with the risk, to be more exposed and to have the slightest habit of caring. The aggressiveness added to the gender culture built the idea of a strong man, has led not to fear the COVID-19. Because social interaction plays an extremely important role for men in normal days, with the arrival of the pandemic I began habits to provide distraction, such as excessive consumption of alcohol and pornography that lead me to sexual compulsion in an uncontrolled manner* (Men’s CSD).

### Central Idea 5: In Subsidence and Retrospection: Neglect and (Dis)Healthcare

Attitudes and practices of neglect and (dis)healthcare are observed in the male discourse in the experience of the COVID-19 pandemic:

*[…] I am not getting used to the isolation and the additional care and restrictions imposed by the authorities. I know it is necessary, but I confess that the situation generated by the pandemic has generated an internal conflict and a great deal of stress, causing me to tend to undue behavior. This is because men are more relapsing in relation to health care. I imagine that this situation should not apply to everyone, but it should reach a significant portion of men, who should not be adapting well to all this, after all the change of habits surprised me unexpectedly and many men just like me needed to change their lifestyle, such as work, physical exercise, leisure and sexual practices. Confess that I neglected the measures, failed to comply with the isolation, stopped wearing the mask and made several meetings with friends during quarantine. I also used medications on my own to prevent infection* (Men’s CSD).

## Discussion

The findings of this study can reveal the deleterious impacts caused to male health arising from the social construction of hegemonic patterns of masculinities that were tensioned by the COVID-19 pandemic in its sociohistorical context.

The strengths of this study are concentrated in the fact that the findings provide insights into an unknown area and provide useful and relevant knowledge about how the pandemic context of COVID-19 has affected men’s lives and health, making it possible to predict its impacts on families and communities, which goes beyond the dimension of transmission and involvement by COVID-19. In addition, it allows advancing the understanding of the social construction of masculinities from a sociohistorical perspective of pointing out the revealing milestones of each event, perception, and male social practice in this place.

The discursive findings are permeated by contradictions, which express the discomfort of men when confronted with tensions to hegemonic patterns of masculinity, driven by the cataclysmic effects caused by the pandemic (Guan et al., 2020; Sousa et al., 2022). The loss of structurally attributed social roles has had significant repercussions on daily routine, the maintenance of status, the performance of tasks and functions, and the way of being and being in the world during a pandemic (Freitas et al., 2020; Lancet, 2020; Nicola et al., 2020). Thus, when experiencing all these events, men have presented responses to coping. In the Brazilian sphere, for example, masculine positions expressed in a massive way, including the presidential representative, incited the adoption of a hegemonic posture of masculinity. The display of messages, such as it’s just a little flu (Lancet, 2020) or we can’t be a sissy’s country, somehow may be influencing the way men are conceiving and dealing with the pandemic phenomenon.

When analyzing how Rosenberg, C. E. (2010) sought to historiographically investigate epidemic disease, it is possible to recognize that the pandemic is permeated by complex characteristic phenomena, which need to be valued, as a way of understanding to cope and know how to face its effects in a less harmful way. In this sense, the framing of the epidemic disease makes it possible to make one realize the denialists movements that surround the emergence of the new disease and that mark the configuration of the first act of the context, to the extent that initially men are confronted with epidemic phenomena, which are generators of conflicts of emotional character in the face of denial and the discomforts generated by masculinities. Thus, how people understand how to manage and establish coping strategies for the current and subsequent pandemic context.

The expanded knowledge about the social phenomenon of the pandemic from the sociohistorical perspective provides contributions to social education in health, the advancement of scientific knowledge about the multiple social dimensions mobilized from an epidemic event. In addition, it allows it to be understood with greater characterization, the way men interact with diseases causing epidemic outbreaks, which can expand the repertoire of social initiatives to be developed with populations.

Regarding the investigated public, it was observed that the existence of strict normative standards of hegemonic masculinity made it difficult to understand what health behaviors men should adopt during the pandemic and caused them to experience conflicts in emotional regulation, with negative reflexes that resulted in the suppression of feelings and disarrangements to mental health driven by emotional instability, in addition to conflicts of decision and male identity. In this direction, the literature has already reinforced the appearance of influences of masculinity norms in male mental health (Milner et al., 2019).

The emergence of significant damage to the mental health status of populations was predictable since the beginning of the epidemic in China (Qiu et al., 2020). Investigations have revealed an important worsening of mental health problems such as anxiety, depression, and increased stress levels and drew attention to the future impacts caused by posttraumatic stress; the emergence of severe mental illness may lead to the risk of suicide (Hiremath et al., 2020).

Consequences relevant to the health sector, such as those imposed by social isolation, were explained in the male discourse, which demonstrated impacts on mood, loss of human contact and the natural environment, increased stress, and the prevalence of Common Mental Disorders (Hiremath et al., 2020; Mamun and Griffiths, 2020; de Sousa et al., 2021; Teixeira et al., 2022).

The standardized practice of the sense of male invulnerability and the adoption of behaviors that are not healthy and even harmful to health were evidenced in the discourse of men, who did not consider themselves in a situation of fragility toward the new disease and now revealed attributes of hegemonic masculinity present in the attitudes and practices constructed and performed. In addition, we found in the discoveries male strategies to deal with the pandemic, which was directed to the harmful conduct of life habits, referring to abusive consumption of alcohol and other drugs, pornographic entertainment, and uncontrolled sexual compulsivity. In this sense, our study’s findings, added to those already evidenced in the scientific literature, reinforce the urgent need to protect male mental health in all life cycles, avoid the harmful effects of machismo on men’s lives, strengthen and create educational initiatives on gender and social construction of masculinities, and the relationship with healthcare with different male audiences, children and adolescents and young people. In addition, a review of public health policies was proposed, with the intention of expanding funding directed to mental health and psychosocial care of the population, as a way to reduce the impacts caused by posttraumatic stress caused by the COVID-19 pandemic.

Healthcare, such as disease prevention and control care, appeared to be discreet and timid for men. Having to adhere to the recommended health measures proved uncomfortable for the public investigated. Such attitudes and practices are close to other health contexts, which reveal that part of the male public neglects and resists the therapies instituted by professionals in health services (Barros et al., 2018; Separavich and Canesqui, 2020).

In the collective discourse of men, the perception of themselves in relation to the emergence of threats to male virility, due to the impairment of affective and sexual routines, impacted the performance of sexual practice, making it limited and causing discomfort, which may be related to the hegemonic pattern of sexual function and the hypersexualization of life (Connell, 2005, 2014; Connell and Messerschmidt, 2013). Thus, it is important to encourage male self-care, such as sexual and reproductive health, self-management, and physical and mental health, as a way to promote post-pandemic resilience and contribute to the increase in health literacy levels, so that men can be more strengthened to deal with the impacts caused by the pandemic and to more effectively face other epidemic events that may arise.

With the threats generated to the symbolic place of family provision, control, and proficiency in public space, added to the fissures caused to economic and financial power, the male discourse revealed discontent with the prolonged permanence in the domestic environment by the performance of new tasks dictated as feminine and the fissures caused to the labor occupation previously exercised and modified by the pandemic. On the contrary, the tensions generated to masculinities may imply reflections and learning, as provided for in the fourth and final act representative of epidemic disease (Rosenberg, C. E., 2010; Oliveira A. C. D. et al., 2020). Therefore, attention is recommended directed to men and their families as an effective strategy to promote bonding, harmony, and the maintenance of family nuclei of support and affection.

In a society structured in patriarchy, colonialism, machismo, and capitalism, certainly the construction of men’s masculinities will be based on references that lead them to imagine and perform attitudes and practices that refer to the ideals of strength, honor, invincibility, domination, control, leadership, and invulnerability, and, in addition, the voracious exercise of virility and sexualization (Connell, 2005, 2014; Connell and Messerschmidt, 2013).

Thus, when questioned or affected by an event or a situation, they can destabilize themselves and suffer more significantly with the possible changes and transformations in the hegemonic model in force. Therefore, they need to be better observed. It is based on these findings found in our study that male behavior in the face of complex social scenarios such as a pandemic can be better understood, enabling the broadening of the therapeutic repertoire of health professionals, qualifying care, strengthening the construction of strategies to protect male mental health, individual and collective growth for post-pandemic resilience, and the overcoming of male attributes harmful to men’s health in their different territories and sociocultural, political and historical contexts (Bühler et al., 2021; Moreira et al., 2021).

Thus, it is recommended to work on the reconfigurations of gender roles in the production of healthcare, whether in institutional spaces or in other social and therapeutic spaces, aiming at overcoming gender inequalities, which impact longitudinally.

Such contexts that permeate the hegemonic attributes of masculinities that are structured in the social context, and which were fissies with the advent of the COVID-19 pandemic, are also an explicit reflection of the epidemic phenomena that make up the second act of the framing of the disease, namely, the possibility of falling ill and dying, social acceptance, the emergence of taboos, cultural and cultural influences, stigma, and discrimination (Rosenberg, C. E., 2010; Rosenberg and Mantovani, 2016). Thus, it is when men recognize the disaggregating potential of the pandemic for themselves, and consequently, for their social construction of masculinity, that these reveal situations representative of the randomness of infection (Rosenberg, C. E., 2010; Rosenberg and Mantovani, 2016).

From the hegemonic masculinity model instituted and socially accepted, social expectations are constructed about the profile of men, hoping that they will be providers of their families, be sexually dominant, present behaviors that involve risks, and have difficulties to demonstrate or discuss their emotions or seek help. This fact is associated with higher rates of addictions, suicide, homicide, and traffic accidents among men, as well as the development of chronic non-communicable diseases such as hypertension, heart problems, and diabetes, among others. Thus, it is necessary to have a close look at health professionals in order to promote actions for male mental health (PAHO, 2019).

It was possible to observe in this study that men now perceive themselves in this hegemonic model of masculinity, and now question it, and recognize that it is provocative of negative repercussions for themselves and for their health. This process of going to come reflective may be evidencing the negotiations that are being made by men regarding the denial and credit of the disease, whether at the individual or collective level, through the responses of society and the community to the perception, meaning and coping with the epidemic disease, which thus configures the third act of the theoretical context of COVID-19 (Oliveira, 2020; Sousa A. R. et al., 2020; Medrado et al., 2021). This act is permeated by public pressures, the emergence of the institution of collective sanitary measures such as vaccination; social distancing; the closure of schools, trades, and industries; and circulation bans (Rosenberg, C. E., 2010; Rosenberg and Mantovani, 2016; Blog do prisco, 2020; Motta, 2020).

Moreover, during the COVID-19 pandemic, denialism in Brazil took alarming proportions, manifesting itself in the denial or minimization of the severity of the disease. In this sense, situating the denialism of the pandemic within a broader phenomenon is fundamental for the action of health educators. This involves unraveling its origin and its relationship with certain political, economic forces, with conservative values, with necropolitics, and also addressing the reasons for its popularization. These are early-stage discussions that allow us to understand and problematize denialism and its contemporary growth (Morel and Massadar, 2021).

Thus, health professionals need to promote educational strategies to reduce uncertainties; promote population adhering to prevention protocols; demonstrate therapeutic treatments without scientific validation; promote vaccination, so as not to compromise the country’s response to the pandemic; and prevent the threat to democracy.

Even though the existence of rigid contours of masculinity among the investigated group is observed, when the collective existence of the sense of invulnerability and the exercise of behaviors harmful to male health are observed, this place can be viewed with positivity, since it is notorious to demonstrate a self-reflective process about itself and its masculinities (Sousa A. R. et al., 2020). In this context, reflections and learning scans in the face of the experience of the pandemic appear late means in this group of men investigated, making the consummation of the fourth act of the context a question not yet given among these individuals. Moreover, Rosenberg draws attention to the early forgetfulness of the disease, without understanding the importance of epidemic events emerging in the dramaturgic daily life of an episodic disease, such as those that generate a pandemic (Silveira and Figueiredo, 2009; Rosenberg, C. E., 2010; Rosenberg and Mantovani, 2016; Blog do prisco, 2020; Motta, 2020; Neto, 2020).

The fact of early forgetting of the pandemic, such as denying it, was observed in the male public in Brazil. Encouraging governmental, non-governmental, and organized civil society actions with a focus on male education for the adoption of a culture of care, which allows to deal responsibly with the epidemic and other events, needs to be a priority among countries. Thus, an intersectoral work, articulated with the different social organizations such as schools, universities, religious denominations, associations, groups, and entities, should be structured in their countries to overcome the disadvantages caused by machismo, which has negatively impacted the ecosystem.

Other relevant aspects need to be reflected in relation to masculinities and the context of the pandemic, such as overcoming individualism and reviewing neoliberal policies and male sex education (Lamb et al., 2021). In countries such the United States, the work done with men in this perspective can bring contributions in government actions; generalize public health approaches; resignify attitudes, beliefs, and individual male responses attributed to the health crisis of COVID-19, and the lessons that can be learned from the pandemic and its intersections with gender (Lamb et al., 2021).

Based on these considerations, it is recommended that the theoretical-political lens of intersectionality can be explored for a better understanding of the processes of social construction of masculinities, their relations with health, disease, and care, especially among groups of men marked by social and health vulnerabilities, such as black men, homosexuals, bisexual, and transgender, and in the context of urban poverty, as evidenced in a study conducted before the advent of the COVID-19 pandemic (Oliveira E. et al., 2020).

If there is no longer the possibility of denying the existence of the disease, since impacts are experienced through the advent of the disease, the latter act is configured characteristically by the subsidence of the outbreak and its retrospection, which can be fast but can also be lasting. Behavioral phenomena can be recognized as an escape, the search for protection, and unavoidable circumstances such as falling ill, dying, surviving, focusing on cases, hospitalizations and deaths, epidemiological fluctuations of indicators, and the evolutionary stage of the epidemiological process.

Moreover, it is in the fourth and final act that the expectations that surround the feeling of change, transformation, overcoming unequal, unjust and distressing contexts, and the realization of new meanings and feelings of a “new normal,” permeated by significant consequences—social, economic, educational, political, cultural, demographic and historical, already dimensioned, corrected, qualified or not (Silveira and Figueiredo, 2009; Rosenberg, C. E., 2010; Rosenberg and Mantovani, 2016; Blog do prisco, 2020; Motta, 2020; Neto, 2020).

Although they are in positions of privilege, men whose masculinities are stuck in hegemonic patterns, the recognition of conflicts in the understanding of what is “if man,” already implies expressive mobilization of the masculinist constructs. Thus, it is possible to infer that the pandemic brought to light the possibility of recognizing the male as to their frailties, even though they are permeated by denial (Rosenberg, C. E., 2010; Rosenberg and Mantovani, 2016), when the need to rethink self-toxic and degrading patterns, and the construction of new references, based on a positive and healthy masculinity.

In this sense, it is necessary to deconstruct the idea that there is a single model of hegemonic masculinity, where there is no room to surface feelings and emotional frailties. This model is mainly associated with negative characteristics, which portray men as non-emotional, independent, non-caregivers, aggressive and non-passionate. These characteristics contribute to the spread of toxic practices, such as physical violence against women and criminal behavior (Connell and Messerschmidt, 2013).

### Implications for Practice and Research

The contributions of the study focus on the expansion and deepening of the sociohistorical analysis of the COVID-19 pandemic, the revealing of the relational dimensions of gender in the experiences of men about the patterns of masculinities expressed, which cause deleterious impacts on male health. In addition, the study allowed us to locate the context of COVID-19 disease from daily, relational and symbolic facts of men’s lives. It provides a substantial basis for studies and practices focused on male health and epidemic diseases. Thus, it dialogs with the need to expand the implementation of public policies, such as the National Policy for Integral Attention to Men’s Health, the Brazilian Ministry of Health, and health professionals and related areas that perform actions for men.

### Study Limitations

The limitations of the study are expressed in the use of a single technique for data collection, which may have generated loss of data seizure, when combined with other techniques, such as those performed face-to-face; the disparate reach of participants in the digital social networks surveyed, which may have concentrated on the sample in specific cycles of ambiences. The measurement of a concentric sample in a region of the country, which may have seized a particular territorial cutout, and the availability of the data collection form only in the virtual ambience. This may have excluded the men who do not have access to online technological resources and/or who do not have skills in the use of Information and Communication Technologies (ICT).

## Conclusion

The male discourse revealed that men have their masculinity structured in the hegemonic model, but express signs of recognition that this model causes harm to themselves and their health. In the experience of the hegemonic model of male, men do not explain the adoption of healthcare attitudes, making them more exposed to the transmission of the new coronavirus, causing COVID-19, and the deleterious effects caused by the pandemic.

The findings allowed us to understand that the progressive revelation of COVID-19 among men was permeated by conflicts in emotional regulation and suppression of feeling in relation to the pandemic, whereas the management and randomness of COVID-19 were marked by a sense of commitment to the family provision and threats to virility that the negotiation of the response to the public mobilized the self-perception of invulnerability and the practice of harmful behaviors, which implied subsidence and careless retrospection with the health of men.

The experience of the pandemic exposed the normative patterns of masculinities from the consummation of acts representative of the pandemic context, which incited men to deny the existence of COVID-19 disease and to delay the understanding and adoption of protection and control measures of COVID-19. As a repercussion; men presented conflicts in the regulation of emotions; presented emotional suppression, were more reactive; felt threatened regarding the loss of the role of family provider, virility; and revealed a sense of invulnerability, added to the weakening of self-care.

Finally, this study contributes to the opening of a field of research aimed at masculinities and the health of men in pandemic contexts. Although it is expected that the pandemic exaggerates health behaviors with unfavorable outcomes among the male public, this study advances in scientific knowledge by explaining the sequence of behavioral events explanatory of the experience experienced by men in a context of the global health crisis through the expression of their masculinities.

## Data Availability Statement

The original contributions presented in the study are included in the article/supplementary material, further inquiries can be directed to the corresponding author/s.

## Ethics Statement

The studies involving human participants were reviewed and approved by Universidade Federal da Bahia. The patients/participants provided their written informed consent to participate in this study.

## Author Contributions

ARS contributed to conception and design of the study and supervised the study. WM, TS, IA, CB, ÉA, MM, RS, JT, LL, NG, ES, ÁFS, LA, LV, and ÁP organized the database, performed the statistical analysis, and wrote the first draft of the manuscript. All authors contributed to manuscript revision, proofread, and approval of the submitted version.

## Conflict of Interest

The authors declare that the research was conducted in the absence of any commercial or financial relationships that could be construed as a potential conflict of interest.

## Publisher’s Note

All claims expressed in this article are solely those of the authors and do not necessarily represent those of their affiliated organizations, or those of the publisher, the editors and the reviewers. Any product that may be evaluated in this article, or claim that may be made by its manufacturer, is not guaranteed or endorsed by the publisher.
